# Is Burnout Infectious? Understanding Drivers of Burnout and Job Satisfaction Among Academic Infectious Diseases Physicians

**DOI:** 10.1093/ofid/ofz092

**Published:** 2019-02-23

**Authors:** Priya Nori, Rachel Bartash, Kelsie Cowman, Melissa Dackis, Liise-anne Pirofski

**Affiliations:** 1 Division of Infectious Diseases, Department of Medicine, Montefiore Medical Center; 2 Department of Psychiatry and Behavioral Sciences, Montefiore Medical Center; 3 Department of Microbiology and Immunology, Albert Einstein College of Medicine, Bronx, New York

**Keywords:** burnout, infectious diseases specialists, job description, job satisfaction

## Abstract

Burnout is pervasive in academic medicine. We administered the Maslach Burnout Inventory and an Infectious Diseases (ID) job description survey to our ID faculty. Respondents’ burnout (>50%) and job satisfaction (>90%) were each high. Although burnout may be balanced by job satisfaction, the relationship between the 2 deserves further study.

Physician burnout is defined as (1) emotional exhaustion, (2) depersonalization, and (3) doubts about competence, accomplishment, and the value of one’s work [1–4]. Burnout is identified as problematic by >96% of survey respondents from all facets of healthcare [3]. Physicians experiencing burnout are more likely to report medical errors and receive lower patient-satisfaction scores [5]. Burnout can lead to physician attrition, which in turn increases workload and dissatisfaction among colleagues, leading to further attrition [6]. Therefore, burnout is “infectious” and healthcare institutions must invest in the well-being of their physician workforce [4–6].

In 1992, Deckard et al [7] queried the infectious diseases (ID) community on the 3 domains of burnout with an Infectious Diseases Society of America-commissioned survey. Although many respondents had high scores on emotional exhaustion (43.5%) and depersonalization (40.3%), the vast majority also reported high personal accomplishment (91.8%) using the Maslach Burnout Inventory (MBI).

Burnout can emanate from caring for marginalized patients [4]. Our patients at the Montefiore Medical Center, Albert Einstein College of Medicine ([MMC] Bronx, NY) have complex medical and mental health comorbidities, significant poverty, and substance abuse. The Bronx remains an epicenter of opioid addiction, human immunodeficiency virus, and hepatitis C [8, 9]. Physicians practicing in such environments are at high risk of exhaustion [5]. Therefore, we were compelled to evaluate burnout among our ID faculty, particularly ID-specific factors affecting both burnout and career satisfaction.

## METHODS

This study was conducted as part of the 2018 MMC ID divisional retreat on burnout, wellness, and stress management. The division consists of faculty who identify their primary job description as physician scientist/funded investigator, clinician, clinician-educator, or hospital administrator, with several having multiple roles. The program agenda included (1) a panel discussion on system and individual approaches to addressing burnout, (2) an energy management coaching session, and (3) a workplace yoga demonstration. Featured speakers included a clinical psychologist and the Chief Wellness Officer at a neighboring institution.

Two voluntary and anonymous surveys were administered during the retreat. An introductory letter served as informed consent. To ensure confidentiality and anonymity, signatures were not required.

### Survey 1

A Maslach Burnout Inventory-Human Services Survey (MBI-HSS) [10], the gold standard burnout assessment tool for healthcare professionals, was given and assessed 3 domains: emotional exhaustion, depersonalization, and lack of personal accomplishment. Participants score statements using a 0–6 frequency scale. Summed scores for each domain were compared with standardized thresholds from the MBI-HSS manual. Overall burnout is defined as a high score in either emotional exhaustion or depersonalization subscales [10].

### Survey 2

The ID Burnout Inventory, designed by our study team, uses a 5-point likert scale to assess stressors specific to primary job descriptions and provides the option to select more than 1 role. The primary study outcome was the prevalence of ID faculty burnout, defined by the MBI-HSS manual [10]. The secondary outcome was identifying specific stressors pertinent to ID faculty. Survey 1 and 2 responses were not linked with each other or to the individual respondent to preserve anonymity.

Bivariate associations were assessed using χ^2^ tests or Fisher’s exact tests where appropriate. All tests were 2-sided with a type 1 error level of 0.05. The study was approved by the MMC Institutional Review Board (no. 2018-9299, July 23, 2018).

## RESULTS

Fifty-one adult and pediatric ID faculty were invited to the retreat, 38 of which attended (excluding 4 study team faculty). Thirty-two (94%) MBI-HSS and 29 (85%) ID Inventories were completed.

Fifteen participants were female (52%) and 25 reported working full-time (86%). Fifteen participants (52%) were over the age of 50. Nine participants (31%) were at their current position for less than 5 years. Primary roles were as follows: 6 administrative, 24 clinical, 7 research, 6 teaching. All faculty completed the clinical, teaching, and universal portions of the ID survey.

Of the MBI-HSS indices, 11 (34%) participants scored highly on emotional exhaustion and 12 (38%) scored highly on depersonalization. Seventeen (53%) participants met criteria for burnout. Fifteen (47%) scored highly on the personal accomplishment subscale. Participants with high personal accomplishment were less likely to meet burnout criteria (24% vs 76%, *P *< .05).

**Figure 1.**
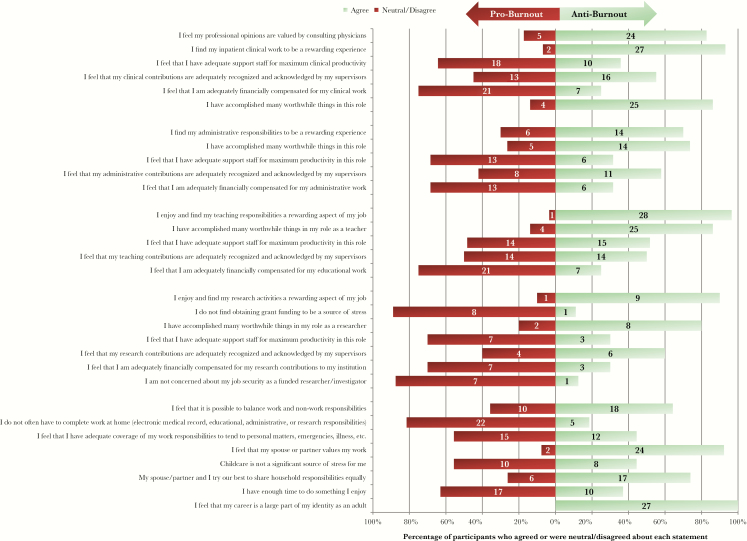
Results from selected infectious diseases-survey items.

The ID inventory results are shown in Figure 1 (full results in Supplemental Table 1). Faculty in all roles felt they had accomplished worthwhile things (86% clinical; 74% administrative, 86% teaching, 80% research) and found their work rewarding (93% clinical; 70% administrative, 97% teaching, 90% research). The majority did not feel they had adequate support staff (64% clinical; 68% administrative, 48% teaching, 70% research) or financial compensation (75% clinical; 68% administrative, 75% teaching, 70% research). In bivariate analysis (Supplemental Table 2), significantly more males felt they had adequate coverage to tend to personal matters (77% male vs 13% female, *P *< .05). More females reported that childcare was a source of stress compared with their male colleagues, although not statistically significant (27% male vs 70% female, *P *= .09). Significantly more faculty under age 50 reported that childcare was a source of stress than those over age 50 (73% <50 years, 20% >50 years; *P *< .05). Significantly more females than males were asked to stop working at home by family (8% male, 64% female; *P *< .05).

Major stressors reported at the retreat and reiterated on ID surveys include the following: challenges with work-life balance, teaching versus billing expectations, prior authorization, electronic medical record constraints, and lack of departmental support for protected scholarly time. As a follow-up activity, faculty voted for a “women in academic medicine” interest group.

## Discussion

In a 2017 Medscape study, ID physicians reported one of the highest burnout rates (55%) [11]. Our study similarly shows that 53% of ID faculty reported burnout. The majority reported stressors similar to prior studies [3], including lack of adequate compensation and support staff for maximum productivity in job-specific roles. Undercompensation is an impediment to attracting trainees to ID and threatens the future of our workforce [12, 13]. Ensuring compensation commensurate to our societal contributions as ID physicians is a worthwhile investment [13].

Despite the high burnout prevalence, approximately half of our faculty scored highly on the personal accomplishment subscale, and >70% felt their work was rewarding regardless of role. Most or all responded, “I feel that my spouse or partner values my work,” and “I feel that my career is a large part of my identity as an adult.” These results suggest that although burnout is prevalent in ID, there is a substantial sense of accomplishment across multiple job-specific roles. Our study mirrors findings of Deckard et [7], which emphasized the coexistence of burnout and accomplishment in ID physicians throughout the country. Although accomplishment may mitigate negative effects of burnout, further study is needed to test this hypothesis.

Several between-group comparisons deserve mention. Significantly more males reported adequate coverage to tend to personal matters, and more females reported that childcare was a source of stress, although not statistically significant. Likewise, significantly more females were asked to stop working at home by family members. These findings underscore the need for healthcare institutions to support the needs of physician parents, particularly women.

We recognize that the scope of our study is limited to ID faculty at a single institution in the Northeast, where physician burnout is prevalent [3]. Nonetheless, our results are similar to a 25-year-old study of ID physicians demonstrating (1) lower scores on emotional exhaustion and depersonalization and (2) higher personal accomplishment reported by academic ID physicians. Authors state that overall “Infectious diseases physicians are generally very satisfied with their jobs and experience a high sense of personal accomplishment”; however, 43.5% also reported a high degree of emotional exhaustion [7]. Expanding on these findings, our ID survey has the potential to identify factors that contribute to and protect against burnout throughout academic medicine. As such, we hope to expand our survey study to other academic divisions at MMC and throughout the country.

To maintain anonymity, MBI and ID inventories were not linked, thereby limiting correlation of MBI scores with job descriptions and gender. However, if conducted on a larger scale, linkage of surveys would provide invaluable information. Administering surveys during a burnout-themed faculty retreat, although potentially introducing recall bias, is an innovative approach to increasing faculty and leadership engagement with this important issue. The retreat enabled faculty to reflect on burnout and practice resiliency strategies as a group. The retreat concluded with a team-building exercise to reflect on our identities as ID educators. To further assess the impact of the retreat, we will re-administer both surveys at our next annual retreat and compare results.

## Conclusions

Meaningful work, strong relationships, positive team structures, and social connection are essential for physician well-being [14]. Our study reiterates that high risk of burnout, along with high rewards from personal and professional accomplishment, are important facets of our careers as ID physicians. Our MBI-HSS results suggest that faculty reporting high accomplishment were less likely to meet burnout criteria; however, further study is needed to understand how job satisfaction, faculty engagement, mentoring, and career development instill a sense of value that may mitigate ID physician burnout. The key to our resiliency and longevity as a profession rests in the value derived from our work, whether from patient care, laboratory breakthroughs, or teaching and mentoring future ID physicians.

## Supplementary Data

Supplementary materials are available at *Open Forum Infectious Diseases* online. Consisting of data provided by the authors to benefit the reader, the posted materials are not copyedited and are the sole responsibility of the authors, so questions or comments should be addressed to the corresponding author.

ofz092_suppl_Supplemental_Tables
