# Supplementary material for: Is Burnout Infectious? Understanding Drivers of Burnout and Job Satisfaction Among Academic Infectious Diseases Physicians
Source: Open Forum Infect Dis. 2019 Feb 23;6(4):ofz092. doi: 10.1093/ofid/ofz092 (PMC6483804; doi:10.1093/ofid/ofz092)
Supplement: ofz092_suppl_Supplemental_Tables [file ofz092_suppl_supplemental_tables.docx]

Supplemental Tables

Table 1: : Results from all ID-survey items

|  | Agree*  n (%) | Missing/NA | Total |
| --- | --- | --- | --- |
| CLINICAL |  |  |  |
| I feel my professional opinions are valued by consulting physicians | 24 (83) |  | 29 |
| I feel my professional recommendations are carried out by consulting physicians | 24 (83) |  | 29 |
| I feel that disagreements with primary providers due to differences of opinion regarding patient care are a source of stress | 13 (45) |  | 29 |
| I find my inpatient clinical work to be a rewarding experience | 27 (93) |  | 29 |
| I find my outpatient clinical work to be a rewarding experience | 21 (81) | 3 | 26 |
| I feel that I have adequate support staff for maximum clinical productivity | 10 (36) | 1 | 28 |
| I have a good understanding of the productivity benchmarks set by my department | 10 (36) | 1 | 28 |
| I feel that my clinical contributions to the department are adequately recognized and acknowledged by my supervisors | 16 (55) |  | 29 |
| I feel that I am adequately financially compensated for my clinical work | 7 (25) | 1 | 28 |
| I have accomplished many worthwhile things in this role | 25 (86) |  | 29 |
| I feel that my clinical work is helping to advance my career | 14 (50) | 1 | 28 |
| In the past month, fatigue has impacted my clinical decision-making | 5 (19) | 2 | 27 |
| ADMINISTRATIVE |  |  |  |
| I enjoy the administrative responsibilities of my job | 14 (70) | 9 | 20 |
| I find my administrative responsibilities to be a rewarding experience | 14 (70) | 9 | 20 |
| I have accomplished many worthwhile things in this role | 14 (74) | 10 | 19 |
| I find my administrative responsibilities expanding over time | 13 (68) | 10 | 19 |
| I feel that I have adequate support staff for maximum productivity in this role | 6 (32) | 10 | 19 |
| I feel that my administrative contributions to my department are adequately recognized and acknowledged by my supervisors | 11 (58) | 10 | 19 |
| I feel that I am adequately financially compensated for my administrative work | 6 (32) | 10 | 19 |
| I feel that my administrative work is helping to advance my career | 7 (39) | 11 | 18 |
| TEACHING |  |  |  |
| I enjoy and find my teaching responsibilities a rewarding aspect of my job | 28 (97) |  | 29 |
| If given a choice, I prefer to be on teaching services, rather than non-teaching services | 21 (81) | 3 | 26 |
| I find my teaching responsibilities expanding over time | 7 (24) |  | 29 |
| I have accomplished many worthwhile things in my role as a teacher | 25 (86) |  | 29 |
| I feel that I have adequate support staff for maximum productivity in this role | 15 (52) |  | 29 |
| I feel that my teaching contributions to my department are adequately recognized and acknowledged by my supervisors | 14 (50) | 1 | 28 |
| I feel that I am adequately financially compensated for my educational work | 7 (25) | 1 | 28 |
| I feel that my educational work is helping to advance my career | 14 (50) | 1 | 28 |
| RESEARCH |  |  |  |
| I enjoy and find my research activities a rewarding aspect of my job | 9 (90) | 19 | 10 |
| I thrive in the atmosphere of funded research | 6 (67) | 20 | 9 |
| I find obtaining grant funding to be a source of stress | 8 (89) | 20 | 9 |
| I feel that teaching responsibilities impede my productivity as a researcher | 2 (22) | 20 | 9 |
| I feel that clinical responsibilities impede my productivity as a researcher | 3 (30) | 19 | 10 |
| My research responsibilities make it difficult to be involved in divisional or departmental activities | 1 (10) | 19 | 10 |
| I have accomplished many worthwhile things in my role as a researcher | 8 (80) | 19 | 10 |
| I feel that I have adequate support staff for maximum productivity in this role | 3 (30) | 19 | 10 |
| I am satisfied with the process of recruiting graduate students/research assistants/fellows to work in my lab | 2 (29) | 22 | 7 |
| I feel that my research contributions to my division/department are adequately recognized and acknowledged by my supervisors | 6 (60) | 19 | 10 |
| I feel that I am adequately financially compensated for my research contributions to my institution | 3 (30) | 19 | 10 |
| I feel that my research work is helping to advance my career | 8 (80) | 19 | 10 |
| I am concerned about my job security as a funded researcher/investigator | 7 (88) | 21 | 8 |
| I’ve experienced disappointment when receiving a poor score on a grant | 7 (88) | 21 | 8 |
| I feel that I’ve let down my lab/research team when we receive a poor score on a grant | 4 (50) | 21 | 8 |
| I find it difficult to recover from a setback in my research | 4 (44) | 20 | 9 |
| UNIVERSAL |  |  |  |
| I feel that it is possible to balance work and non-work responsibilities | 18 (64) | 1 | 28 |
| I have a good understanding of milestones in my primary work role and the path to achieving these | 20 (74) | 2 | 27 |
| I would like to be more involved with research but I don’t have the time | 12 (48) | 4 | 25 |
| I would like to be more involved with research but I don’t know how to pay for it | 14 (58) | 5 | 24 |
| I often have to complete work at home (EMR, educational, administrative, or research responsibilities) | 22 (81) | 2 | 27 |
| I feel that I have adequate time off from work to tend to my personal health and well-being | 7 (26) | 2 | 27 |
| I feel that I have adequate coverage of my work responsibilities to tend to personal matters, emergencies, illness, etc. | 12 (44) | 2 | 27 |
| I feel that my spouse or partner values my work | 24 (92) | 3 | 26 |
| I often receive work calls at night or on the weekends | 17 (65) | 3 | 26 |
| I have been asked by family members to stop working at home | 10 (37) | 2 | 27 |
| Childcare is a significant source of stress for me | 10 (56) | 11 | 18 |
| My spouse/partner and I try our best to share household responsibilities equally | 17 (74) | 6 | 23 |
| I have enough time away from work and family/personal responsibilities to do something I enjoy (self-care, exercise, reading, hobbies, etc.) | 10 (37) | 2 | 27 |
| I try my best to use all of my vacation days | 15 (56) | 2 | 27 |
| I have taken work calls/meetings/emails while I am on vacation | 26 (96) | 2 | 27 |
| I feel that my career is a large part of my identity as an adult | 27 (100) | 2 | 27 |

*Includes participants who agreed or strongly agreed to each statement.

**Table 2: Bivariate analyses**

|  | Female | Male | p-value* | ≤50 years old | >50 years old | p-value* |
| --- | --- | --- | --- | --- | --- | --- |
| Accomplishment in role (clinical) | 11 (73) | 14 (100) | 0.10 | 12 (86) | 12 (87) | >0.99 |
| Compensation (clinical) | 3 (21) | 4 (28) | >0.99 | 4 (29) | 3 (21) | >0.99 |
| Adequate Clinical Support staff | 6 (43) | 4 (28) | 0.70 | 7 (50) | 3 (21) | 0.24 |
| Clinical contributions recognized | 7 (47) | 9 (64) | 0.46 | 8 (57) | 8 (53) | >0.99 |
| Work-life balance | 8 (57) | 10 (71) | 0.23 | 8 (57) | 10 (71) | 0.23 |
| Adequate coverage | 2 (13) | 10 (77) | <0.001^†^ | 6 (43) | 6 (46) | >0.99 |
| Childcare is source of stress | 7 (70) | 3 (27) | 0.09 | 8 (73) | 2(20) | 0.03^†^ |
| Family members asked to stop working at home | 9 (64) | 1 (8) | <0.001^†^ | 7 (50) | 3 (23) | 0.24 |
| My partner shares household responsibilities | 9 (69) | 8 (62) | >0.99 | 8 (67) | 9 (64) | >0.99 |
| My partner values my work | 11 (85) | 13 (100) | 0.48 | 11 (85) | 13 (100) | 0.48 |
| I have time for self-care | 3 (21) | 7 (54) | 0.12 | 3 (21) | 7 (54) | 0.12 |
| I try to use all vacation days | 8 (57) | 7 (53) | >0.99 | 8 (57) | 7 (54) | >0.99 |
| Often work from home | 11 (79) | 11 (85) | >0.99 | 11 (79) | 11 (85) | >0.99 |
| Often work on vacation | 13 (93) | 13 (100) | >0.99 | 13 (93) | 13 (100) | >0.99 |

*Calculated using chi square test or Fisher’s exact test, where appropriate.

^†^significant at .05 level.
